# Diffuse Large B-Cell Lymphoma in an Adolescent Male Presenting as Ureteral Stricture

**DOI:** 10.1155/2014/239345

**Published:** 2014-06-29

**Authors:** Christopher D. Jaeger, Kelly L. McAlvany, Shannon N. Zingula, Stephen A. Kramer, Candace F. Granberg

**Affiliations:** ^1^The Department of Urology, Mayo Clinic School of Medicine, 200 1st Street SW, Rochester, MN 55905, USA; ^2^The Department of Radiology, Mayo Clinic School of Medicine, 200 1st Street SW, Rochester, MN 55905, USA

## Abstract

Lymphoma may affect the ureter in cases of retroperitoneal involvement. We present a case of an adolescent male found to have non-Hodgkin lymphoma initially presenting as ureteral stricture evident on imaging. He was treated and responded to multiagent chemotherapy with resolution of both the lymphoma and the ureteral stricture. Although rare, non-Hodgkin lymphoma should be included in the differential diagnosis of pediatric patients with noncalculous, idiopathic ureteral strictures.

## 1. Introduction

While the initial clinical presentation of non-Hodgkin lymphoma (NHL) varies based on lymphoma type and location, diffuse large B-cell lymphoma (DLBCL) may present with a rapid worsening of focal symptoms from nodal or extranodal disease, consistent with other diffuse lymphomas [1]. We present a unique case of an adolescent found to have non-Hodgkin lymphoma initially presenting as ureteral stricture.

## 2. Case Presentation

**Figure 1:**
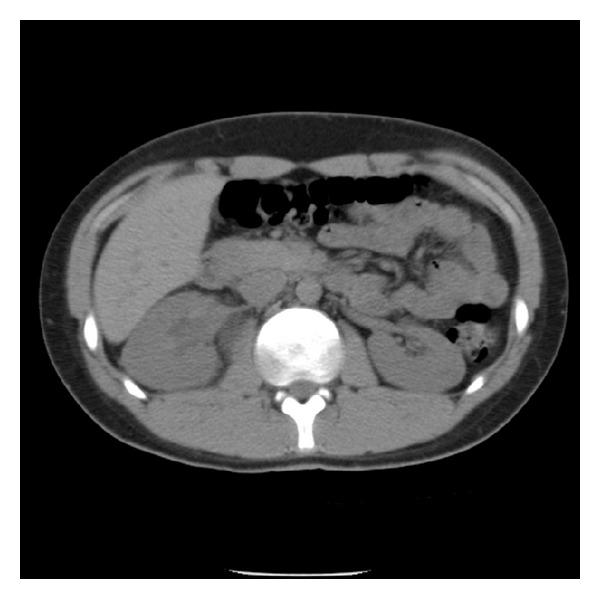
Axial CT image demonstrating nonspecific soft tissue density along the right perinephric space without hydronephrosis.

**Figure 2:**
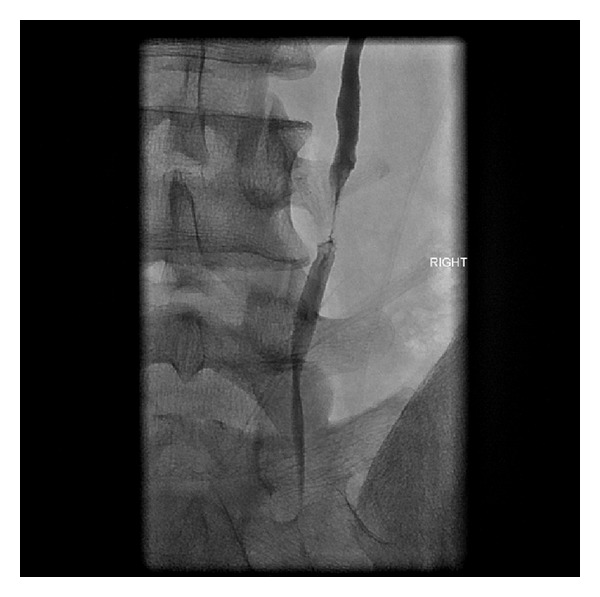
Nephrostogram demonstrating midureteral stricture.

**Figure 3:**
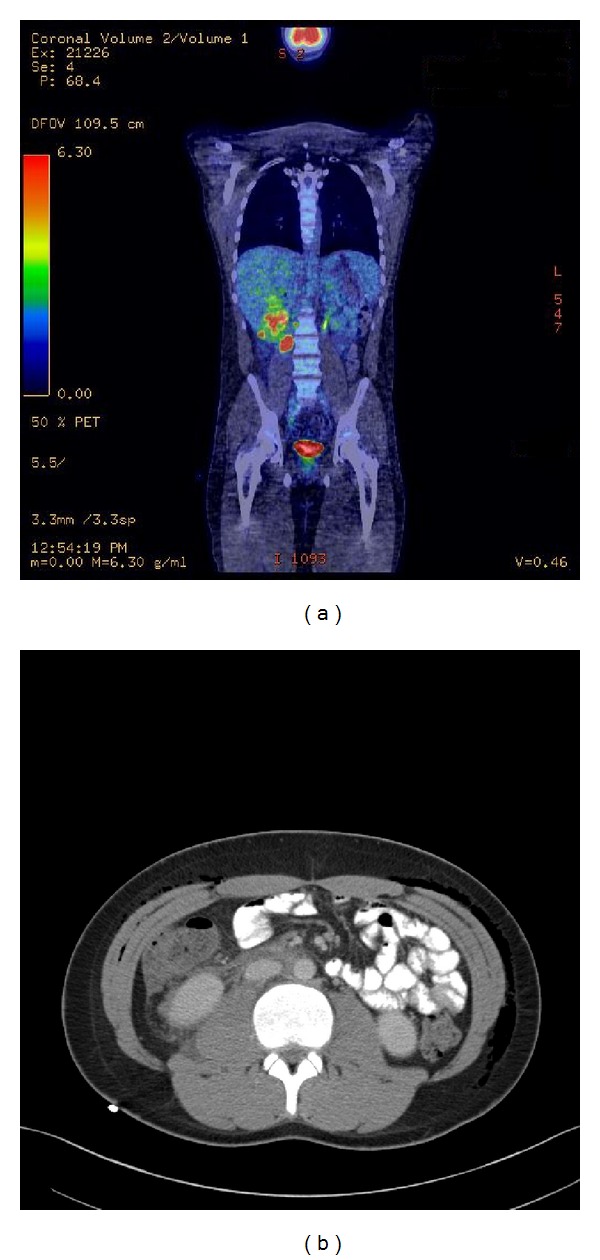
Coronal PET-CT and contrast CT images demonstrating PET-avid areas suspicious for lymphoma and vague infiltrative soft tissue process in the retroperitoneum.

**Figure 4:**
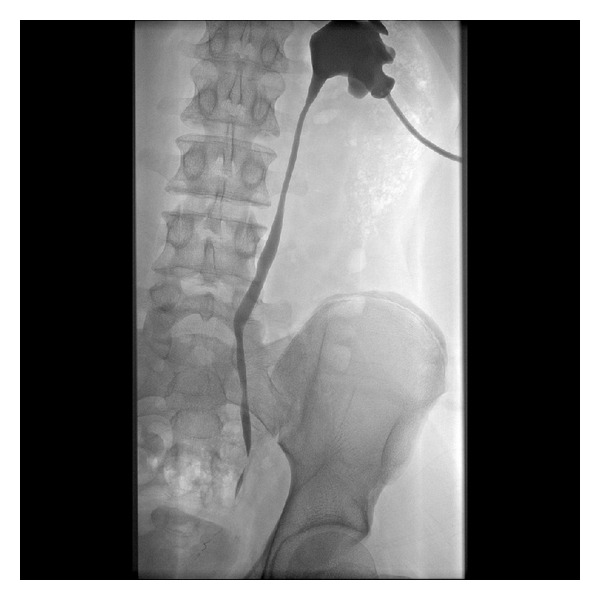
Follow-up nephrostogram demonstrating resolution of stricture after chemotherapy.

A 16-year-old male was referred with subacute-onset right flank pain with hematuria. Initial CT scan without contrast (Figure 1) obtained at an outside institution demonstrated no evidence of urolithiasis, lymphadenopathy, or hydronephrosis. Subsequent imaging, including repeat noncontrast CT scan, intravenous pyelogram, and retrograde pyelogram, demonstrated no evidence of ureteral obstruction, decreased renal function, collecting system displacement, or other pathologies. A trial of indwelling right ureteral stent was performed for unexplained pain without improvement and thus removed after one week. The referring surgeon noted difficulty in passing the stent. The patient represented three months later with worsening flank pain. Ultrasonography demonstrated hydronephrosis. Nuclear medicine renogram with furosemide revealed decreased function of the right kidney (15%) without response to diuretic, consistent with obstruction. A retrograde pyelogram demonstrated a two-centimeter midureteral stricture. A ureteral stent was left indwelling. Upon presentation, the patient underwent nephrostomy tube placement with ureteral stent removal and antegrade nephrostogram demonstrated midureteral narrowing (Figure 2) as noted at the time of retrograde pyelogram. Findings were thought to be due to a traumatic stent placement earlier in the patient's clinical course. The patient ultimately underwent robot-assisted exploration for ureteral stricture repair at which time significant fibrotic tissue was found encasing the right ureter. Biopsy with frozen section pathologic analysis demonstrated lymphoma. Final pathology demonstrated large lymphoid cells with immunoperoxidase studies demonstrating expression for CD10, CD20, bcl-6, and LMO2, consistent with DLBCL. Subsequent staging with positron emission tomography-computed tomography (PET-CT) showed intense PET-avid infiltrative soft tissue in the right perinephric space and retroperitoneum without significant adenopathy, and contrast CT confirmed infiltrative soft tissue attenuation along the retroperitoneum (Figure 3). The patient was initiated on induction chemotherapy per protocol ANHL01P1 (off-study) with vincristine, prednisone, high-dose and intrathecal methotrexate, cyclophosphamide, and doxorubicin following reduction treatment and noted complete radiographic response [2]. Subsequent nephrostogram demonstrated complete resolution of the ureteral obstruction (Figure 4), and the nephrostomy tube was removed after well-tolerated clamping trials.

## 3. Discussion

Lymphomas are the third most frequent cancer in children, with DLBCL being the most common subtype of NHL in adolescents [3]. In the pediatric population, NHL is often aggressive and of high grade. Initial presentation varies depending on subtype and involved area, with signs including enlarging palpable lymphadenopathy, nonspecific fever, weight loss, or night sweats (so-called B symptoms), or symptoms secondary to compression of adjacent structures, often within the neck, abdomen, or mediastinum. This is a unique case of DLBCL involving the ureter that, to our knowledge, has not previously been reported in the pediatric or adolescent literature.

Lymphomas directly involving the urinary tract are rare, although a compressive effect secondary to retroperitoneal lymphadenopathy is present more often than invasion or primary extranodal organ involvement. Ureteral obstruction exists in multiple reports with variable incidence of less than 1% to 14% and only a minority of patients experiencing symptoms of obstruction [4–6]. When displaced by lymphoma, the ureter classically deviates laterally near the ureteropelvic junction and medially as the ureter traverses the pelvic brim. Encasement of the ureters by NHL may cause functional obstruction without radiographic dilation of the collecting systems [7]. Notably, the patient's flank pain was present prior to radiographic evidence of obstruction, and only persistent symptoms warranted repeat imaging, perhaps consistent with this previously reported observation of obstruction in the absence of collecting system dilation.

Lymphomas arising in the genitourinary tract account for less than 5% of extranodal lymphomas [8]. While cases of extranodal isolated ureteral lymphoma have been reported, the most common genitourinary site for extranodal involvement is the kidney. Of all subtypes, DLBCL is the most common subtype to present in this fashion. Ureteric involvement was noted in 16% of patients in an autopsy series by Scharifker, with eight of 21 patients having ureteral infiltration by lymphomatous tissue [6]. As direct visualization within the ureteral lumen itself was not accomplished in this case, we cannot comment on whether the patient's lymphoma was isolated to external compression or if there was a degree of invasion into the wall, although the latter would be consistent with the patient's history of hematuria. Fortunately, systemic therapy resulted in complete response and resolution of the ureteral stricture.

## 4. Conclusion

We report an interesting case of NHL presenting as a ureteral stricture in an adolescent male. Although rare, NHL should be included in the differential diagnosis of the adolescent patient with noncalculous, idiopathic ureteral stricture.

## References

[1] Anderson T, Chabner BA, Young RC (1982). Malignant lymphoma. I. The histology and staging of 473 patients at the National Cancer Institute. *Cancer*.

[2] Goldman S, Smith L, Anderson JR (2013). Rituximab and FAB/LMB 96 chemotherapy in children with stage III/IV B-cell non-Hodgkin lymphoma: a children's oncology group report. *Leukemia*.

[3] Percy C, Smith M, Linet M, La R (1999). Lymphomas and reticuloendothelial neoplasms. *Cancer Incidence and Survival among Children and Adolescents: United States SEER Program 1975–1995*.

[4] Rosenberg SA, Diamond HD, Jaslowitz B (1961). Lymphosarcoma: a review of 1269 cases. *Medicine*.

[5] Abeloff MD, Lenhard RE (1974). Clinical management of ureteral obstruction secondary to malignant lymphoma. *Johns Hopkins Medical Journal*.

[6] Scharifker D, Chalasani A (1978). Ureteral involvement by malignant lymphoma. Ten year's experience. *Archives of Pathology and Laboratory Medicine*.

[7] Chong BH, Trew P, Meng L, Pitney WR (1981). Anuric renal failure due to encasement of the ureters by lymphoma. Ureteric obstruction without dilatation. *Australian and New Zealand Journal of Medicine*.

[8] Schniederjan SD, Osunkoya AO (2009). Lymphoid neoplasms of the urinary tract and male genital organs: a clinicopathological study of 40 cases. *Modern Pathology*.

